# The epidemiology of *Plasmodium vivax* and *Plasmodium falciparum* malaria in China, 2004–2012: from intensified control to elimination

**DOI:** 10.1186/1475-2875-13-419

**Published:** 2014-11-03

**Authors:** Qian Zhang, Shengjie Lai, Canjun Zheng, Honglong Zhang, Sheng Zhou, Wenbiao Hu, Archie CA Clements, Xiao-Nong Zhou, Weizhong Yang, Simon I Hay, Hongjie Yu, Zhongjie Li

**Affiliations:** Division of Infectious Diseases, Key Laboratory of Surveillance and Early-warning on Infectious Disease, Chinese Center for Disease Control and Prevention, 155 Changbai Road, Changping District, Beijing 102206 China; School of Public Health and Social Work, Queensland University of Technology, Brisbane, Australia; Research School of Population Health, College of Medicine, Biology and Environment, The Australian National University, Canberra, Australia; National Institute of Parasitic Diseases, Chinese Center for Disease Control and Prevention, Ruijin 2nd Road, Shanghai, China; Spatial Ecology and Epidemiology Group, Tinbergen Building, Department of Zoology, University of Oxford, South Parks Road, Oxford, OX1 3PS UK; Fogarty International Center, National Institutes of Health, Bethesda, MD 20892 USA; Key Laboratory on Biology of Parasite and Vector, Ministry of Health, WHO Collaborating, Shanghai, China

**Keywords:** Malaria, Epidemiology, Control, Elimination, China

## Abstract

**Background:**

In China, the national malaria elimination programme has been operating since 2010. This study aimed to explore the epidemiological changes in patterns of malaria in China from intensified control to elimination stages.

**Methods:**

Data on nationwide malaria cases from 2004 to 2012 were extracted from the Chinese national malaria surveillance system. The secular trend, gender and age features, seasonality, and spatial distribution by *Plasmodium* species were analysed.

**Results:**

In total, 238,443 malaria cases were reported, and the proportion of *Plasmodium falciparum* increased drastically from <10% before 2010 to 55.2% in 2012. From 2004 to 2006, malaria showed a significantly increasing trend and with the highest incidence peak in 2006 (4.6/100,000), while from 2007 onwards, malaria decreased sharply to only 0.18/100,000 in 2012. Males and young age groups became the predominantly affected population. The areas affected by *Plasmodium vivax* malaria shrunk, while areas affected by *P. falciparum* malaria expanded from 294 counties in 2004 to 600 counties in 2012.

**Conclusions:**

This study demonstrated that malaria has decreased dramatically in the last five years, especially since the Chinese government launched a malaria elimination programme in 2010, and areas with reported falciparum malaria cases have expanded over recent years. These findings suggest that elimination efforts should be improved to meet these changes, so as to achieve the nationwide malaria elimination goal in China in 2020.

## Background

Malaria, transmitted via the bites of *Anopheles* mosquitoes, is one of the most important parasitic diseases affecting mankind [1]. More than 3.4 billion people were exposed to the risk of malaria in 2012, and there were an estimated 207 million cases and 627,000 malaria deaths [2, 3]. Much effort and progress to control and eliminate malaria has been made by the World Health Organization, affected countries as well as many other cooperative partners [4]. By 2010, 108 countries in the world are malaria free and 99 countries have continuing malaria transmission [5].

Malaria had a wide geographical distribution in China. Both *Plasmodium falciparum* and *Plasmodium vivax* malaria have been historically present, with a high incidence. Before the foundation of the People’s Republic of China in 1949, it was estimated that 30 million malaria cases occurred yearly and 70% of the counties were endemic for malaria [6, 7]. Since then, organizations for control and scientific malaria research have been established and large-scale surveys and anti-malarial campaigns carried out among the regions with high transmission of malaria. These initiatives included strengthening the case reporting system, improving access to treatment, preventive anti-malarial administration for the high-risk groups, environmental improvement, vector control, and social mobilization [8, 9]. More recently, annual numbers of reported malaria cases have declined significantly through years of effort, to less than 26,000 by 2000 [10]. The Chinese government implemented intensive interventions between 2000 and 2010, and the national malaria elimination programme was launched from 2010, with the National Action Plan for Malaria Elimination being issued officially in 2010 [11]. An unprecedented fall in numbers of malaria cases was observed subsequently [12–14]. Currently, little research has been conducted on the changes of malaria epidemiologic features from the control stage (pre-2010) to the elimination stage (2010 onwards) in China [15]. In this paper, the epidemiological characteristics of both *P. vivax* and *P. falciparum* malaria in China were described during this transition period, so as to help prioritize current and future resource allocation for malaria elimination.

## Methods

### Data collection

In this study, the nationwide data on malaria cases were obtained from 2004 to 2012 from the national malaria surveillance system, hosted by the Chinese Center for Disease Control and Prevention. In China, malaria is a statutory notifiable infectious disease, and cases are identified according to the unified diagnostic criteria issued by the Chinese Ministry of Health, which includes the definitions of clinically diagnosed and laboratory-confirmed cases. Clinically diagnosed cases were defined as a patient with malaria-like symptoms, having lived in or recently travelled to areas with known malaria transmission. Laboratory-confirmed cases were defined as clinically diagnosed cases with any of the following lab test results relating to malaria: malaria parasites confirmed by microscopy, rapid diagnostic tests (RDTs), or polymerase chain reaction test [16, 17]. Health workers in both the public and private medical sectors were required to report malaria cases. Both clinically diagnosed and laboratory-confirmed cases were included in this study. The species *P. falciparum* and *P. vivax* were further identified.

A standard form was adopted by local physicians and epidemiologist to collect individual information on each malaria case, including age, gender, address, date of onset, diagnosis, and laboratory test result. Routine case and malaria-associated death reporting is done by hospitals. Population data for every province in China from 2004 to 2012 were retrieved from the National Bureau of Statistics of China.

### Seasonal feature analysis

Seasonal index was used to understand seasonal patterns of malaria incidence. The index was calculated by month, and it was the average case number for a given month (i.e. May) divided by the mean of case number in that corresponding month (i.e. May) during the whole nine years of 2004-2012 [18]. No obvious seasonal pattern was expected if the seasonal index of each month was close to 1.0 [18].

### Geographic distribution of disease incidence

Annual malaria incidence rate for *P. vivax* and *P. falciparum* was calculated by dividing the number of annual cases by the corresponding population, and multiplying by 100,000. The incidence rate of every year by province was mapped to present the geographic distribution of malaria. Numbers of total counties and newly affected counties for each species of malaria were calculated separately every year. The total counties were defined as the cumulative number of counties where malaria had been detected by year, and the counties with malaria cases occurring for the first time since 2004 were considered as newly affected counties in the corresponding year. These counties were then regarded as existing affected areas in the following years. The software ArcGIS version 9.1 was used to describe the spatial distribution of malaria using a county-level polygon map.

## Results

### Overall epidemic trend

During the nine years of 2004 to 2012, 238,443 cases of malaria were reported in China, with 184,118 (77.2%) cases of *P. vivax* malaria, 17,878 (7.5%) cases of *P. falciparum* malaria, and 36,447 (15.3%) other cases, which included 28 (0.01%) cases of *Plasmodium ovale* malaria, 52 (0.02%) cases of *Plasmodium malariae* malaria, 108 (0.04%) mixed infection cases and 36,259 (15.2%) untyped cases (Table 1). Before 2010, <10% of all malaria cases were caused by *P. falciparum*. However, this increased substantially following 2010, reaching 55.2% in 2012.

**Table 1:** **The cases and death of malaria by plasmodium species in China, 2004–2012**

Characteristic	2004	2005	2006	2007	2008	2009	2010	2011	2012	Total
Cases										
Overall	37537	39656	60193	46673	26358	14098	7389	4088	2451	238443
*P. vivax* (%)	27410 (73.0)	30399 (76.7)	47687 (79.2)	38441 (82.4)	21168 (80.3)	10678 (75.7)	4901 (66.3)	2414 (59.1)	1020 (41.6)	184118 (77.2)
*P. falciparum* (%)	3616 (9.6)	3711 (9.4)	2804 (4.7)	1648 (3.5)	996 (3.8)	1033 (7.3)	1288 (17.4)	1429 (35.0)	1353 (55.2)	17878 (7.5)
Others* (%)	6511 (17.3)	5546 (14.0)	9702 (16.1)	6584 (14.1)	4194 (15.9)	2387 (16.9)	1200 (16.2)	245 (6.0)	78 (3.2)	36447 (15.3)
Death										
Overall	32	45	34	14	22	10	14	30	14	215
*P. vivax* (%)	3 (9.4)	2 (4.4)	4 (11.8)	4 (28.6)	3 (13.6)	0 (0)	0 (0)	0 (0)	0 (0)	16 (7.4)
*P. falciparum* (%)	29 (90.6)	43 (95.6)	30 (88.2)	10 (71.4)	19 (86.4)	10 (100)	14 (100)	30 (100)	14 (100)	199 (92.6)

*Others contained *Plasmodium ovale*, *Plasmodium malariae*, mixed infection cases and untyped cases. The numbers in the brackets were the proportions of cases or deaths by plasmodium species in overall number.

Overall, 215 deaths from malaria were reported, most of which (92.1%) were caused by *P. falciparum* (Table 1). One hundred and ninety-one fatal cases (88.9%) were male, and the mean age of deaths was 35.4 years (range: 4–85 years). The crude fatality rate of all cases was 0.09%, with the lowest being 0.03% in 2007 and the highest being 0.73% in 2011. The overall fatality rate of *P. falciparum* was 1.11% with the lowest recorded in 2007 (0.61%) and the peak in 2011 (2.10%). The overall fatality rate of *P. vivax* was 0.01%, and no deaths from *P. vivax* were reported since 2009.

**Figure 1:**
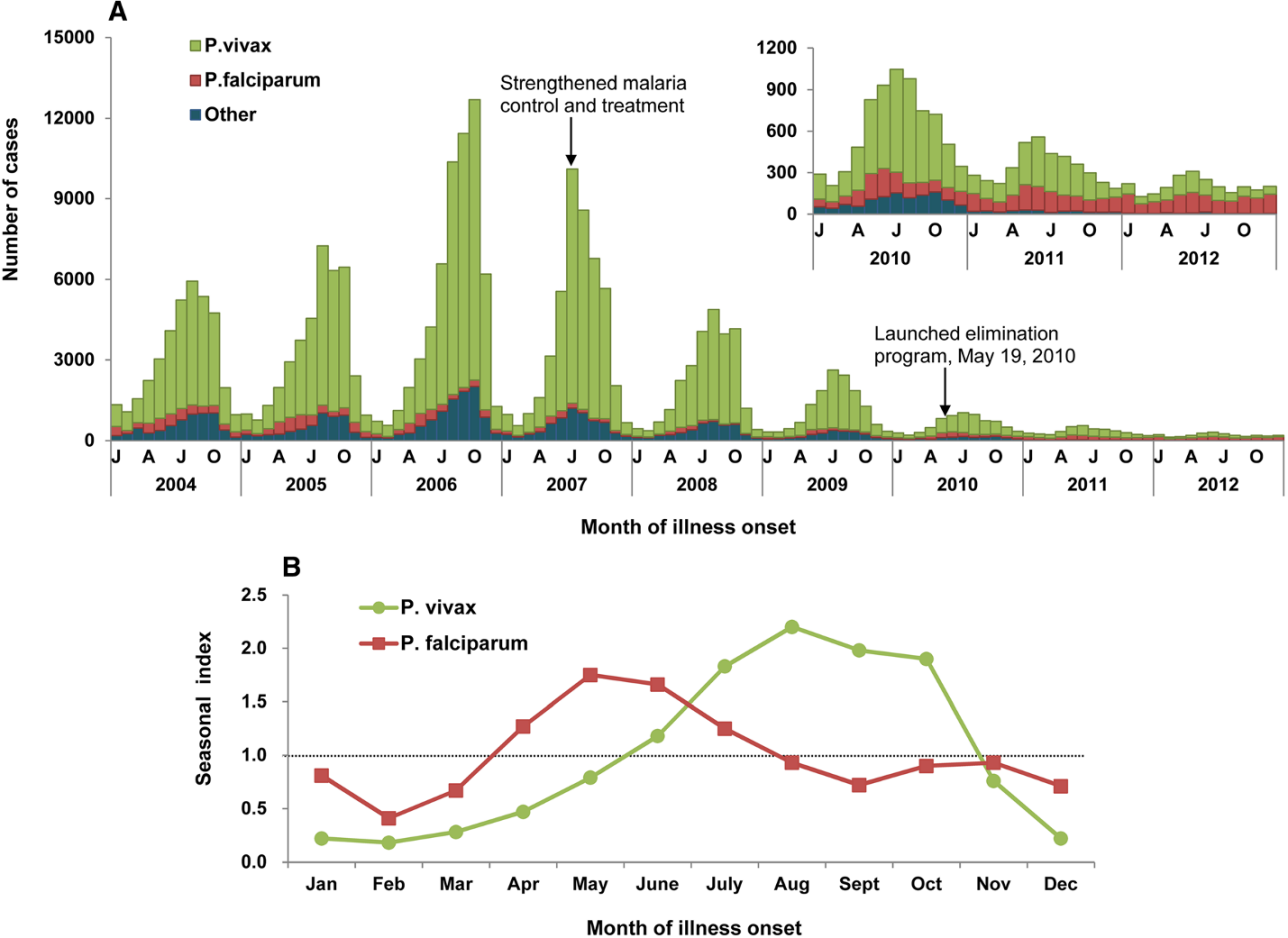
**The seasonal distribution of malaria cases by month in China, 2004–2012. (A)** The epidemic curve of cases by plasmodium species. Others contained *Plasmodium ovale*, *Plasmodium malariae*, mixed infection cases and untyped cases. **(B)** The seasonal index of P. vivax and P. falciparum malaria. The index was calculated by month, and it was the average case number for a given month (i.e. May) divided by the mean of case number in that corresponding month (i.e. May) during the whole nine years of 2004-2012. No obvious seasonal fluctuation was expected if the seasonal index of each month was close to 1.0.

The average annual incidence rate was 2.0 cases/100,000 during the whole nine years. Annual incidence rate presented striking variation by year, with the highest recorded in 2006 (4.6/100,000) and the lowest level in 2012 (0.18/100,000). Table 1 and Figure 1A showed that *P. vivax* malaria increased continuously from 2004 to 2006, with annual incidence rate of 2.11-3.65 cases/100,000, peaking in 2006, and then decreasing sharply from 2007 to 2012, with an annual incidence rate of 0.08-2.92 cases/100,000. *P. falciparum* malaria was relatively stable from 2004 to 2006, with an annual incidence rate of 0.21-0.29 cases/100,000, then decreasing in 2007 to a rate that has since remained stable at 0.08-0.13 cases/100,000.

### Demographic features

**Figure 2:**
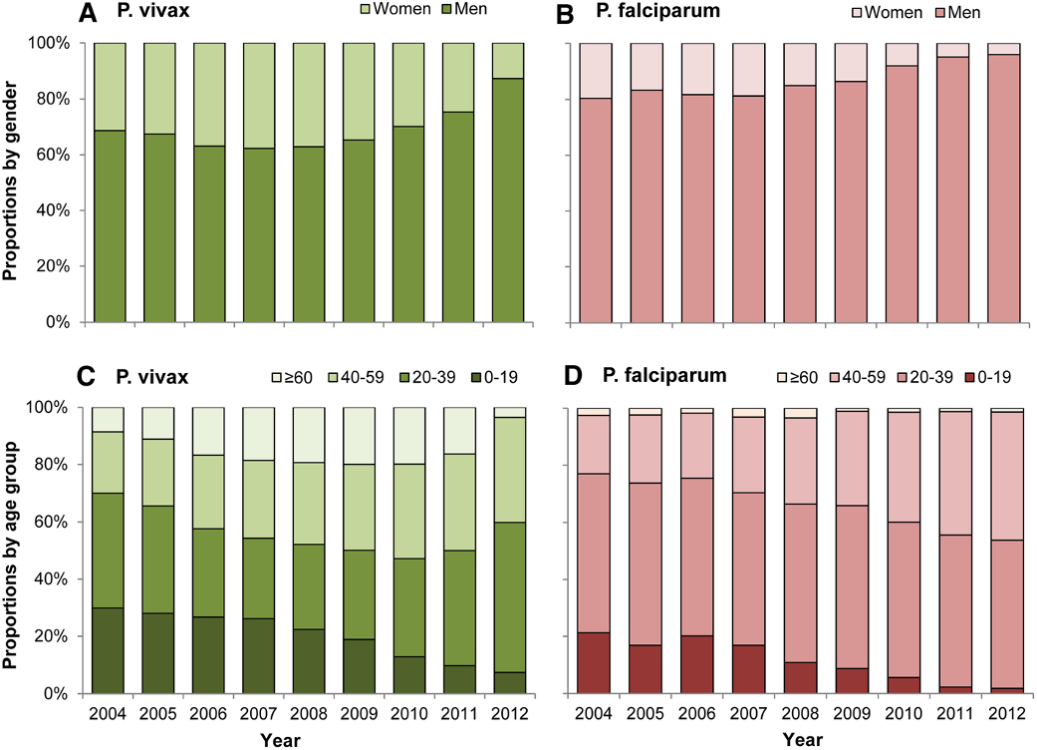
**The gender and age distribution of** ***P. vivax*** **and** ***P. falciparum*** **malaria in China, 2004–2012. (A)** The proportion of cases by gender of *P. vivax* malaria. **(B)** The proportion of cases by gender of *P. falciparum* malaria. **(C)** The proportion of cases by age group of *P. vivax* malaria. **(D)** The proportion of cases by age group of *P. falciparum* malaria.

There was a male predominance among cases of both *P. vivax* and *P. falciparum* malaria, with male-to-female ratios of 1.9:1 and 5.7:1 respectively. The proportion of male patients had risen significantly during recent years (Figure 2A and B). For *P. falciparum* malaria, the male-to-female ratio reached 23.6:1 in 2012.

Figure 2C and D showed that the proportion of malaria cases among the 0–19 age group decreased during the study period, with the proportion of *P. vivax* malaria occurring among this age group dropping from 30% in 2004 to 7.5% in 2012, and for *P. falciparum* malaria from 21% in 2004 to 2% in 2012. By contrast, the proportion of cases among 20–59 year-olds increased for *P. vivax* (from 62% in 2004 to 89% in 2012) and *P. falciparum* (from 76% in 2004 to 97% in 2012).

### Seasonal features

*Plasmodium vivax* and *P. falciparum* malaria presented obvious seasonal characteristics during the study period (Figure 1B). *Plasmodium falciparum* malaria occurred most frequently in May-June, during which 28% of cases were reported. The seasonal index was the highest (1.8) and the average monthly incidence rate was 0.022/100,000 in May. A relatively high incidence rate of *P. vivax* malaria was observed during July-October, during which 66% of cases were reported. The average monthly incidence rate reached the peak in August (0.29/100,000) and the seasonal index was 2.2.

For *P. vivax,* the epidemic season occurred a little earlier in the period 2007–2009 than 2004–2006, and no definite seasonal trend was observed in 2010–2012. For *P. falciparum* in 2004–2006, a primary peak occurred during the months of April–June, with a secondary peak in October-December. The primary peak became weaker in 2010–2012 and the secondary peak nearly vanished in 2007–2012 (Figure 1A).

### Geographical distribution

**Figure 3:**
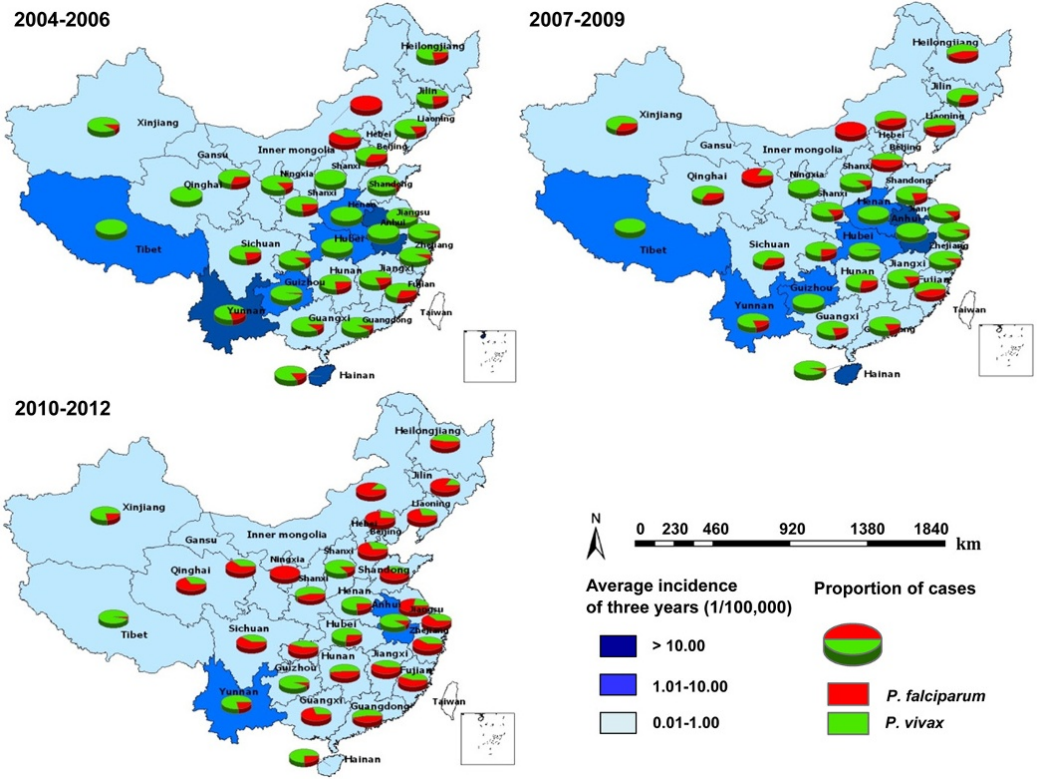
**The geographical distribution of** ***P. vivax and P. falciparum*** **malaria by year in China, 2004–2012.**

Figure 3 shows the geographical distribution of malaria incidence rate, including the proportion of cases infected with *P. vivax* and *P. falciparum*, by province. All 31 provinces in mainland China reported malaria cases. The number of provinces with malaria incidence rate of ≥10/100,000 reduced from 3 (Hainan, Anhui and Yunnan, with dark blue color) in 2004–2006, to 2 (Hainan and Anhui) in 2007–2009, to none in 2010–2012.

The proportion of cases caused by *P. vivax* and *P. falciparum* changed markedly in each of the 31 provinces during 2004–2012. From 2004–2006, *P. vivax* malaria predominated in 29/31 provinces, with only Inner Mongolia and Beijing, located in northern of China, having more *P. falciparum* than *P. vivax* malaria. From 2010–2012, areas with *P. vivax* shrunk markedly and *P. falciparum* became the predominant species among 18/31 provinces (Figure 3), mostly in the north, east and south of China. Several central regions and border provinces (Heilongjiang, Xinjiang, Tibet, and Yunnan province) still had more *P. vivax* malaria cases than *P. falciparum* in 2010–2012.

**Figure 4:**
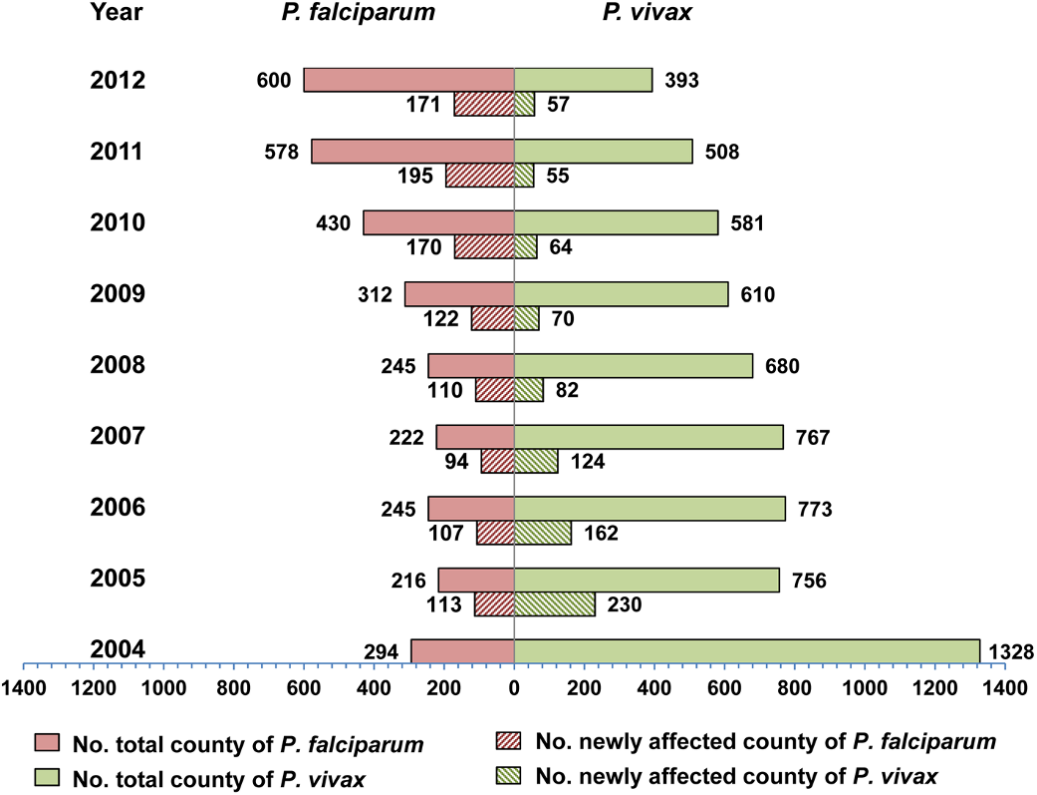
**The changing of counties affected by** ***P. vivax and P. falciparum*** **malaria in China, 2004–2012.**

Figure 4 showed that the cumulative number of counties and newly affected counties with *P. vivax* malaria declined greatly: numbers of affected counties decreased from 1328 (46% of all counties in China) in 2004 to 393 (14%) in 2012; and numbers of newly affected counties decreased from 230 in 2005 to 57 in 2012. By contrast, the cumulative number of counties with *P. falciparum* malaria increased from 294 in 2004 to 600 in 2012; and the number of newly affected counties increased from 113 in 2005 to 171 in 2012.

## Discussion

This study, utilizing a longitudinal surveillance dataset spanning 9 years, demonstrated the great changes occurring with respect to the epidemiological characteristics of malaria in China during a period of transition from intensified control to elimination. The incidence rate of *P. vivax* and *P. falciparum* both decreased dramatically in the past decade, especially from 2010 when the Chinese government launched a national malaria elimination programme. However, the number of areas with *P. falciparum* malaria has increased dramatically during the study period.

Three epidemiologic stages were observed during the study period: malaria cases increased from 2004–2006, decreased from 2007–2009, and kept at a very low, stable level from 2010–2012. A total of 60,193 malaria cases were reported in 2006, which was the highest number of malaria cases in the 21^st^ century. This was mainly caused by the re-emergence of malaria in the region along the Huanghuai River of central China, especially in Anhui and Henan Provinces. Changing meteorological factors, resulting in increasing vectorial capacity and basic reproductive rate of *Anopheles sinensis* may lead to further malaria re-emergence in these areas [19–22]. Malaria in these affected areas was effectively controlled by the strengthened Anti-Malaria Programme in 2007, which included several intensified anti-malaria measures, including active screening for malaria cases in high-risk villages, providing greater access to treatment for *P. vivax* malaria cases facilitated by case tracing and recording of treatment history, preventive administration of anti-malarial medicines in high-risk populations, including people who lived in the same household as malaria cases, and conducting vector control in the high transmission areas [23–25].

The demographic characteristics of malaria cases changed markedly, with the predominance of males and young adults increasing during the study period. Migration from the economically backward areas to developed areas, as well as going abroad for business, has exploded since the last decade. It has been suggested that young migrant workers, who leave their original community to another place for work, likely comprise a high-risk group for malaria in China [26]. Most young migrant workers live in the suburbs of the cities. Poor living conditions and a lack of anti-mosquito facilities likely increase opportunities to be exposed to the vector mosquitoes [26]. A total of 215 deaths from malaria were reported, which maintained at a relatively low level during nine years. The fatality rate increased slightly in recent years, which could possibly be attributed to the sharp decrease of cases occurring and the delayed diagnosis of imported malaria cases, as more and more cases existed in the previously non-epidemic areas and the clinicians lacked awareness of malaria diagnosis. The research on malaria fatalities in China and its determinants should be further conducted.

The seasonal analysis demonstrated that peak activity of *P. vivax* malaria occurred later (July–October) than *P. falciparum* (May–June). China is a country with a vast territory and contains several climatic zones, and the two types of malaria had different epidemic areas, where meteorological factors and dominate vectors vary greatly, which may explain the difference of seasonal features of the two types of malaria. Along with the rapid reduction in malaria incidence and more sporadic cases among the returners from overseas countries, seasonal variation of cases also lessened [27, 28]. It may be a reason for weakened seasonal fluctuation of these two types of malaria epidemics during recent years.

Although incidence rate of *P. vivax* malaria declined significantly after the strengthened anti-malaria programme in 2007, incidence rate of *P. falciparum* malaria stabilized, albeit at a lower level, becoming the dominant subtype in 2012. More than 50% of all malaria cases were caused by *P. falciparum.* What is more, the areas affected by *P. falciparum* malaria expanded greatly over recent years. The increasing trend of imported malaria from overseas during recent years was the probable reason for the geographic expansion of *P. falciparum* malaria*.* One study revealed that many malaria cases were imported in 2011, most of which were caused by *P. falciparum* [13]. In recent years, with the sharp increase in overseas financial investment, global travel and export of labour, more and more people travelled to Africa and Southeast Asia, where *P. falciparum* malaria is highly endemic. According to the statistics from the Chinese Bureau of Immigration, the number of international travellers has grown steadily over the decade, and more than 80 million people left the country to seek job opportunities, travel or study in Africa and Southeast Asia in 2012 [29]. Due to a lack of awareness and poor implementation of measures to prevent malaria infection, many travellers suffer from a high risk of malaria infection [30–32]. Most recent imported cases returned from African countries (*i.e.* Ghana and Angola) or Southeast Asian countries (*i.e.* Myanmar) [33, 34]. Therefore, intensive surveillance and management of exported labour, with timely detection, diagnosis and appropriate treatment, should be prioritized [35]. Training of local epidemiologists and physicians on malaria case diagnosis and investigation, especially in the non-epidemic areas, needs to be enhanced. The epidemiological features of imported malaria cases and its impact on malaria elimination in China should also be explored with long-term data in future research.

This study has two possible limitations. First, not every case could be distinguished as being indigenous or imported during the earlier part of the study period, because the data were not available. Only since the national malaria elimination programme launched in 2010, each malaria cases was then required to be investigated and identified as indigenous or imported. Second, the existence of the untyped cases before 2007 might impact the observed distribution of malaria by plasmodium species. However, the proportion of the untyped cases had decreased sharply with the improvement of capacity of malaria testing after 2007.

## Conclusions

The incidence of malaria in China has decreased dramatically in the past few years, especially after 2010 when the Chinese government launched a national malaria elimination program. However, areas with *P. falciparum* malaria have expanded. Many sporadic cases appeared in the non-epidemic areas, which has brought new challenges for malaria diagnosis and treatment at a local level. Malaria elimination efforts should be further improved to meet these new challenges, so as to effectively allocate public health resources to achieve the goal of national malaria elimination in 2020.
